# Flavin-Containing Monooxygenase 3 Genetic Variants and Possible Susceptibility to Coronary Heart Disease Among Han Chinese With Type 2 Diabetes

**DOI:** 10.1155/ije/1020054

**Published:** 2025-09-23

**Authors:** Yuanmin Mao, Nan Gu, Xiaowei Ma, Yuxin Wang, Na Yu, Difei Lu, Linchao Tong, Xiaohui Guo, Junqing Zhang, Ying Gao, Dahong Yu, Jianping Li

**Affiliations:** ^1^Department of Endocrinology, Peking University First Hospital, Beijing, China; ^2^Department of Cardiology, Peking University First Hospital, Beijing, China

**Keywords:** coronary heart disease, flavin-containing monooxygenase 3, single-nucleotide polymorphisms, type 2 diabetes

## Abstract

**Purpose:** To explore the flavin-containing monooxygenase 3 (FMO3) single-nucleotide polymorphisms (SNPs) and their connection to coronary heart disease (CHD) among Han Chinese with type 2 diabetes (T2D).

**Methods:** The case-control research involved 781 individuals with T2D: 506 CHD cases and 275 controls. The tag-SNPs rs2266780, rs1736557, rs1800822, and rs909530 were selected according to the e!Ensembl database. The genotypes of all the research populations were analyzed via mass spectrometry. SPSS 25.0 software was used to analyze the associations between the selected SNPs and the risk of developing CHD.

**Results:** The rs1800822 T allele frequency was lower in the CHD group than in the non-CHD group (*p* = 0.049), as was the rs909530 T allele frequency (*p* = 0.029). The carriers of rs909530 CX genotype had a greater risk of developing CHD than did the TT genotype carriers in the non-premature CHD group (*p* < 0.001).

**Conclusion:** Our study revealed that rs1800822 and rs909530 in the FMO3 gene may be related to CHD risk among Han Chinese with T2D. We observed a significant gene-by-age interaction at rs909530 on CHD risk, indicating that aging modulates the effect of this locus. Young patients with T2D and the CX genotype may require more stringent management of cardiovascular risk factors.

## 1. Introduction

The 2021 International Diabetes Federation report indicates approximately 536.6 million people worldwide have diabetes. China still ranks first in the world, with approximately 140.9 million people [1]. Despite a decreasing trend in incidence, coronary heart disease (CHD) remains the major complication of type 2 diabetes (T2D). Genetic variation contributes to the risk of T2D and CHD [2].

Flavin-containing monooxygenase (FMO) metabolizes drugs, dietary-derived substances, and environmental pollutants through catalytic activity [3]. The FMO gene family encodes five distinct protein isoforms [4]. The FMO3 enzyme is the primary isoform in the human liver. In the intestine, choline and similar compounds are metabolized by microbes, generating trimethylamine (TMA). FMO3 oxidizes TMA to trimethylamine-N-oxide (TMAO) [5]. The FMO3 gene is located on chromosome 1q23-q25 and encompasses 26,919 bp and contains nine exons (e!Ensembl database GRCh38.p13, https://www.ensembl.org). A 532 amino acid polypeptide is encoded by FMO3 [6]. FMO3 gene expression is modulated by many transcription factors and steroid hormones [7]. Insulin sensitivity and lipid metabolism are significantly influenced by FMO3. In insulin-resistant mice, FMO3 knockdown reduces Forkhead box O1, a metabolic regulatory node, ameliorating hyperglycemia, hyperlipidemia, and atherosclerosis [8]. In mice fed a cholesterol-rich diet, FMO3 knockdown reduced biliary lipid release, intestinal lipid absorption, and hepatic oxysterol and cholesteryl ester synthesis [9]. In addition, FMO3 knockdown can also suppress the expression of inflammatory markers [10]. Moreover, the expression of FMO3 affects TMAO formation, which is significant in the development of atherosclerosis and may act as an indicator for cardiovascular risk [11].

Genetic variations in FMO3 are associated with numerous diseases. People with FMO3 gene mutations that impair FMO3 production or function may develop trimethylaminuria (TMAU) [12]. The rs2266782 variant of the FMO3 gene was linked to a higher incidence of hypertension among smokers in a Russian population [13]. The FMO3 variant Glu158Lys, also Glu308Gly, was linked to the susceptibility to hypertension-related cerebrovascular disease in Turks [14]. A cohort of Italian children carrying four minor alleles across the FMO3 polymorphisms rs2266780 and rs2266782 exhibited a reduced risk of obesity [15]. We speculate that the genetic variants in FMO3 are linked to the risk of developing CHD in T2D patients.

## 2. Methods

### 2.1. Participants

**Figure 1:**
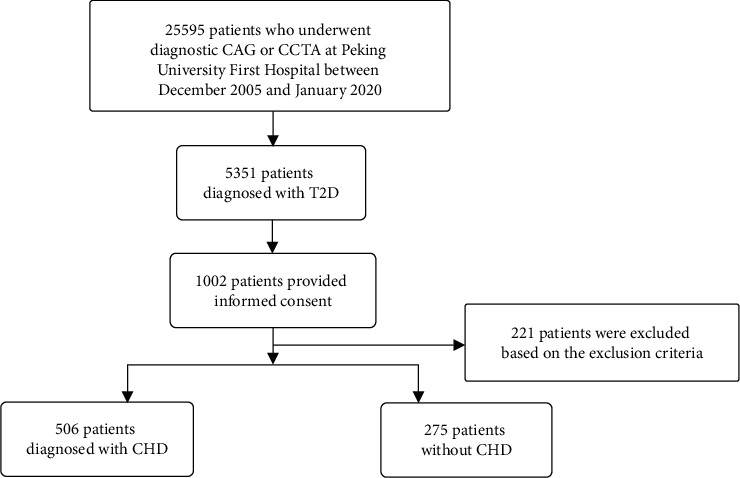
Flowchart of patient screening.

Between December 2005 and January 2020, 781 unrelated participants from Peking University First Hospital (PKUFH) were enrolled (Figure 1).

Inclusion criteria: (1) Diagnosed T2D [16] and (2) coronary angiography (CAG) or coronary computed tomography angiography (CCTA) was performed. Exclusion criteria: (1) other types of diabetes; (2) pregnant women; (3) patients with inflammatory conditions and autoimmune diseases; (4) patients with hematological or solid tumors; (5) patients with prior myocardial infarction and coronary revascularization; and (6) patients with congestive heart failure.

After strict screening, patients were categorized into the CHD group (*n* = 506) and the non-CHD group (*n* = 275). CHD group was defined by diameter stenosis ≥ 50% in at least one major coronary artery branch. In the control group, stenosis of all major coronary arteries was < 50% [17].

### 2.2. Tag-Single-Nucleotide Polymorphisms (SNP) Selection

The Han Chinese in Beijing, China (CHB) data in the e!Ensembl database (GRCh38.p13) (https://www.ensembl.org) was used. The recruitment criteria of tag-SNPs were as follows: (1) Genetic variants were validated in the Han Chinese cohort; (2) minor allele frequency > 0.05; (3) the linkage disequilibrium threshold of *r*^2^ ≤ 0.8 (performed using Haploview 4.2); and (4) classification as tag-SNPs. Ultimately, four tag-SNPs (rs2266780, rs1736557, rs1800822 and rs909530) for the FMO3 gene were selected. They are all located within exons, including three missense variants and one synonymous variant.  Table 1 displays their specific attributes.

### 2.3. DNA Testing

Blood genomic DNA was isolated according to the procedure [18]. They were genotyped via MassARRAY technology (Beijing Compass Biotechnology Co., Ltd., Beijing, China), and the primers were as follows (Table 2). For the four SNPs of the FMO3 genes, the detection rates were all > 99%; 5% of the samples for each SNP were randomly selected for repeated genotype verification, and the results were verified.

### 2.4. Data Analysis

SPSS 25.0 (IBM) was employed to perform data analysis. We employed the independent samples *t*-test, the Mann–Whitney *U* test, and the chi-square (χ^2^) test to analyze clinical characteristics. The associations between the risk of developing CHD and the presence of the selected genotypes were analyzed via χ^2^ tests. We used logistic regression to adjust for covariates and calculated odds ratios (ORs) with 95% confidence intervals (CIs) to assess genotype-outcome associations. Adjustments were made for potential confounders, including age, sex, body mass index (BMI), duration of diabetes, smoking status, high-density lipoprotein cholesterol (HDL-C), low-density lipoprotein cholesterol (LDL-C), estimated glomerular filtration rate (eGFR), and glycated hemoglobin A1c (HbA1c). We set statistical significance at *p* < 0.05.

## 3. Results

### 3.1. Baseline Characteristics of the Study Subjects

As shown in Table 3, cases and controls showed comparable duration of diabetes, hypertension history and triglyceride (TG) levels (*p* > 0.05); however, they differed significantly in sex, age, HbA1c, eGFR, total cholesterol (TC), LDL-C, HDL-C, BMI, fasting blood glucose (FBG), and smoking status (*p* < 0.05).

### 3.2. Association of FMO3 SNP Gene Variants With CHD Risk in T2D Patients

**Figure 2:**
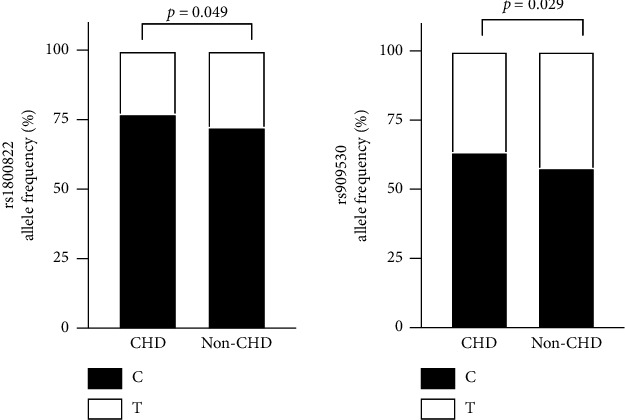
Allele frequency of FMO3 SNPs in the cases and controls.

All four tag-SNPs complied with Hardy–Weinberg equilibrium in controls (*p* > 0.05). The frequency of minor allele T at rs1800822 and rs909530 in CHD patients was lower than that in control participants (OR = 0.787, 95% CI 0.619–0.999, *p*=0.049 and OR = 0.789, 95% CI 0.637–0.976, *p*=0.029, respectively) (Figure 2), and the allele frequencies of rs2266780 and rs1736557 showed no significant differences between cases and controls (data not shown). The genotype frequencies in cases and controls were similar (Figure S1). According to the dominant inheritance model, at rs1800822, carriers of the CT/TT genotype had a lower CHD risk than CC carriers did (OR = 0.742, 95% CI 0.552–0.999, *p*=0.049). Nonetheless, this association lost statistical significance after covariate adjustment (OR' = 0.641, 95% CI 0.366–1.125, *p*=0.121, Table 4). In the recessive and additive inheritance model, at rs909530, TT homozygotes had a lower CHD risk than CC/CT carriers did before we adjusted for other covariates (OR = 0.665, 95% CI 0.452–0.979, *p*=0.038 and OR = 0.619, 95% CI 0.405–0.974, *p*=0.027, respectively), but adjustment for risk factors showed no substantial difference between the two groups. (OR' = 0.698, 95% CI 0.333–1.462, *p*=0.503 and OR' = 0.765, 95% CI 0.342–1.713, *p*=0.515, respectively, Tables S1 and S2).

### 3.3. Linkage Disequilibrium Estimations Between FMO3 SNPs and Haplotype Analysis

We analyzed haplotypes to further explore the gene-disease association. The four variants (rs2266780, rs1736557, rs1800822, and rs909530) were in linkage disequilibrium. The haplotype distributions were comparable between the cases and controls (Table S3).

### 3.4. Stratified Analysis and Test for Interaction

Since the connection between certain SNPs and the risk of getting CHD is significantly influenced by BMI, age, and smoking status [19], a stratified analysis was performed according to the above confounding variables.

Among people with a normal weight (BMI < 24 kg/m^2^), the frequency of allele T at rs909530 in CHD patients was lower than that in controls (OR = 0.547, 95% CI 0.330–0.906, *p*=0.018; data not shown). Neither overweight/obese individuals (BMI ≥ 24 kg/m^2^) nor subgroups stratified by smoking status showed significant case-control differences in allele frequencies or genotype distributions at the four SNPs. No interactive effects of BMI or smoking status on the associations between the SNPs and the risk of developing CHD were detected. Below we present the results of age-stratified analysis and age-genotype interactions.

According to the definition of premature CHD, people were divided into two groups to study the relationships between age and genetic factors in people with T2D. Male patients < 55 years old and female patients < 65 years old were included in the younger group, and male patients ≥ 55 years old and female patients ≥ 65 years old were included in the older group.

In the older group, significant differences in sex, age, smoking status, and metabolic profiles were detected between the cases and controls. Further analysis revealed that the frequency of allele T at rs1800822 in CHD patients was lower than that in non-CHD patients (OR = 0.670, 95% CI 0.492–0.913, *p*=0.011; data not shown). The T allele at rs909530 exhibited a decreasing trend in CHD patients compared to controls, though without reaching statistical significance (OR = 0.772, 95% CI 0.582–1.024, *p*=0.072; data not shown). No significant genotype differences were observed between cases and controls in either age group.

**Figure 3:**
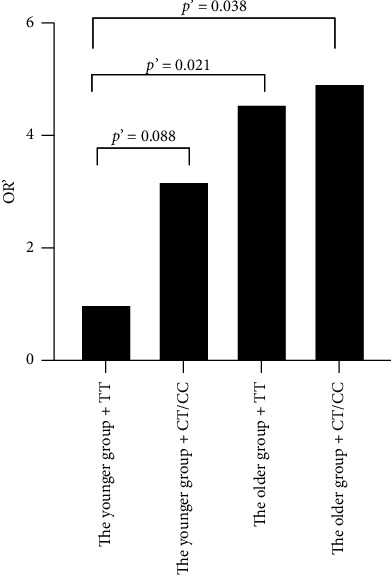
Interaction effect of age and rs909530 on CHD risk.

We then regarded people in the younger group with TT homozygotes as a baseline and used logistic regression analysis to assess the interactive effects. Finally, we observed that people in the older group were clearly more likely to suffer from CHD when they carried the CX genotype after we adjusted for other related factors at rs909530 (TT carriers: OR = 3.000, 95% CI 1.418–6.347, *p*=0.004; OR′ = 4.563, 95% CI 1.251–16.645, *p*=0.021; CX carriers: OR = 4.472, 95% CI 2.370–8.439, *p* < 0.001; OR′ = 4.931, 95% CI 1.094–22.221, *p*=0.038; Figure 3). At rs1800822, people in the older group without TT homozygotes had the greatest risk of having CHD before we adjusted for related factors (OR = 3.978, 95% CI 1.240–12.758, *p*=0.02).

## 4. Discussion

CHD is the main macrovascular complication of T2D and the foremost cause of mortality among diabetic individuals [20]. FMO3 can reduce glucose tolerance, promote lipid metabolism disorders and atherosclerosis formation, and inhibit energy metabolism, thus promoting the occurrence of obesity, and promote the occurrence of inflammation and platelet aggregation [21]. FMO3 produces TMAO, which is intimately associated with the risk of developing metabolic disease and CHD. FMO3 affects disease via routes that are dependent or independent of TMAO. We focused on the variation in FMO3 SNPs and revealed its influence on the risk of developing CHD in T2D patients.

In our study, the rs1800822 and rs909530 T alleles were considerably lower in the CHD group than in the controls. Regarding genotype frequencies at rs1800822 and rs909530, the initial associations between the variants and CHD risk under different inheritance models lost statistical significance after covariate adjustment. We have employed stepwise logistic regression analysis to adjust for covariates. The results revealed that the loss of statistical significance was primarily due to the adjustment for confounding factors (Tables S4–S6). Then, we conducted age-stratified analyses and assessed age-genotype interactions. We found that the carriers of the rs909530 CX genotype had a greater risk of developing CHD than did the TT genotype carriers in the older group. The result demonstrated a significant gene-by-age interaction at rs909530, whereby the CX genotype was associated with a markedly increased risk of CHD specifically in older adults (OR′ = 4.931, *p*′ = 0.038). Research has demonstrated that rs909530 CX genotype carriers had considerably higher liver FMO3 protein abundance than TT genotype carriers. This study also reported a positive correlation between age and the abundance of FMO3 protein [22]. We hypothesize that rs909530 and age may work together to determine the susceptibility to CHD by modulating the amount of the FMO3 protein. The FMO3 protein directly regulates the metabolism of glucose, lipids, and inflammatory factors, altering the risk of developing CHD. High levels of TMAO are a predictor for greater cardiovascular mortality. One study indicated that the SNP rs2266782 in the FMO3 gene affects outcomes in chronic heart failure patients by altering plasma TMAO concentrations, with the AA genotype linked to reduced TMAO levels [9]. The SNP rs2266782 is not in linkage disequilibrium with our four SNPs. However, we cannot rule out the possibility that rs1800822 and rs909530 of FMO3 may also be related to the plasma concentration of TMAO, thus influencing the risk of developing CHD.

There were several limitations in this study. First, we could not obtain enough liver or serum samples to explore the FMO3 protein abundance and TMAO level. Second, this study was conducted at one center with a limited sample size; therefore, selection bias for patients may have occurred. Third, some non-CHD patients were diagnosed via CCTA. A prospective study evaluated the efficacy of CCTA in diagnosing CHD using CAG as the criterion standard. The results demonstrated that the sensitivity of CCTA was 99%, specificity was 64%, positive predictive value was 86%, and negative predictive value was 97% [23]. It demonstrated that a negative CCTA result is highly reliable, while a positive result is less reliable. Therefore, in our study, patients with negative CCTA results were classified into the non-CHD group, while those with positive CCTA results were not included in the CHD group. Nevertheless, to minimize the potential misclassification of patients with mild CHD, we performed a sensitivity analysis by excluding non-CHD patients diagnosed by CCTA (*n* = 102), retaining only those confirmed by CAG (*n* = 173). We found that the inclusion or exclusion of non-CHD patients diagnosed by CCTA did not significantly affect the statistical outcomes (Tables S7–S10). To increase the sample size, we ultimately included non-CHD patients diagnosed by CCTA into the study. Lastly, causal associations cannot be concluded because of the cross-sectional observational approach of this study. Prospective studies and fundamental medical research are needed. The effects of risk variant genes on gene transcription, translation, or posttranslational protein modification need to be further studied.

## 5. Conclusion

Our study revealed that rs1800822 and rs909530 in the FMO3 gene may be related to CHD risk among Han Chinese with T2D. We observed a significant gene-by-age interaction at rs909530 on CHD risk, indicating that aging modulates the effect of this locus. Young patients with T2D and the CX genotype may require more stringent management of cardiovascular risk factors.

## Figures and Tables

**Table 1:** Characteristics of FMO3 tag-SNPs.

tag-SNPs	Allele: frequency (count)	Location	Variant type
rs2266780	A:0.830 (171) G:0.170 (35)	1q24.3 exon7	Missense variant
rs1736557	G:0.723 (149) A:0.277 (57)	1q24.3 exon6	Missense variant
rs1800822	C:0.816 (168) T:0.184 (38)	1q24.3 exon4	Synonymous variant
rs909530	C:0.665 (137) T:0.335 (69)	1q24.3 exon7	Missense variant

**Table 2:** Primers of FMO3 tag-SNPs.

tag-SNPs	PCR forward/reverse primer (5′-3′)
rs2266780	ACGTTGGATGTGGCATTGTGTCCGTAAAGC
	ACGTTGGATGCCTCAAATATGGTCCCATCC

rs1736557	ACGTTGGATGTACCGACAGCCATCTCTGAC
	ACGTTGGATGGGCATCAAGCCATAGTTTTC

rs1800822	ACGTTGGATGGAATCGGCTGTCTTTGATGC
	ACGTTGGATGTGGCCTTACCTGGAAAGGAC

rs909530	ACGTTGGATGGGACCAATAAAACAAGAGGG
	ACGTTGGATGCACAGAATGCTTGCTGGGAG

**Table 3:** Clinical characteristics of the CHD group and non-CHD group.

	CHD (*n* = 506)	Non-CHD (*n* = 275)	*p* value
Sex, male *n* (%)	323 (63.80)	111 (40.36)	**< 0.001**
Age (years)	64.93 ± 9.89	62.30 ± 9.87	**< 0.001**
Diabetes duration (years)	8.00 (5.00–15.00)	8.00 (4.00–16.00)	0.600
Hypertension *n* (%)	378 (74.70)	134 (77.50)	0.468
FBG (mmol/L)	7.24 (6.15–8.83)	6.65 (5.80–7.90)	**0.002**
HbA1c (%)	7.20 (6.60–8.30)	6.80 (6.20–7.65)	**0.003**
eGFR (mL/min/1.73 m^2^)	75.00 ± 22.56	80.34 ± 21.72	**0.022**
TG (mmol/L)	1.61 (1.08–2.33)	1.49 (1.03–2.36)	0.738
TC (mmol/L)	3.82 (3.27–4.65)	4.20 (3.56–4.99)	**0.001**
LDL-C (mmol/L)	2.17 (1.70–2.85)	2.40 (1.85–2.99)	**0.025**
HDL-C (mmol/L)	0.95 (0.83–1.08)	1.05 (0.91–1.25)	**< 0.001**
BMI (kg/m^2^)	26.18 ± 3.67	26.91 ± 3.98	**0.035**

*Note:* Continuous data with normal distribution are shown as means ± SD, skewed data as medians (interquartile ranges), and categorical variables as counts (%). The bold values are the numbers with *p* values less than 0.05.

**Table 4:** Association analysis of FMO3 candidate SNP genotypes in the patients and control participants (dominant inheritance model).

SNP	Genotype	CHD *n* (%)	Non-CHD *n* (%)	OR (95% CI)	*p* value	OR′ (95% CI′)	*p*′ value
rs2266780	AA	360 (71.7)	189 (69.2)	1.000		1.000	
	AG + GG	142 (28.3)	84 (30.8)	1.127 (0.816–1.555)	0.468	1.246 (0.669–2.320)	0.489

rs1736557	GG	300 (63.4)	183 (69.3)	1.000		1.000	
	GA + AA	173 (36.6)	81 (30.7)	0.768 (0.556–1.059)	0.107	1.716 (0.914–3.223)	0.093

rs1800822	CC	298 (59.0)	140 (51.7)	1.000		1.000	
	CT + TT	207 (41.0)	131 (48.3)	0.742 (0.552–0.999)	**0.049**	0.641 (0.366–1.125)	0.121

rs909530	CC	210 (41.7)	98 (36.2)	1.000		1.000	
	CT + TT	294 (58.3)	173 (63.8)	1.261 (0.930–1.710)	0.135	0.961 (0.546–1.691)	0.889

*Note:* The bold values are the numbers with *p* values less than 0.05.

## Data Availability

Data are available upon reasonable request, subject to privacy and ethical restrictions.

## Supporting Information

Figure S1. Association analysis of FMO3 candidate SNP genotype frequencies in the patients and control participants.

Table S1. Association analysis of FMO3 candidate SNP genotypes in the patients and control participants (recessive inheritance model).

Table S2. Association analysis of FMO3 candidate SNP genotypes in the patients and control participants (additive inheritance model).

Table S3. Analysis of haplotype.

Table S4. Association of rs1800822 genotypes with CHD risk in the patients and control participants (dominant inheritance model).

Table S5. Association of rs909530 genotypes with CHD risk in the patients and control participants (recessive inheritance model).

Table S6. Association of rs909530 genotypes with CHD risk in the patients and control participants (additive inheritance model).

Table S7. Clinical characteristics of the cases and controls.

Table S8. Association analysis of FMO3 candidate SNP genotypes in cases and controls (dominant inheritance model).

Table S9. Association analysis of FMO3 candidate SNP genotypes in cases and controls (recessive inheritance model).

Table S10. Association analysis of FMO3 candidate SNP genotypes in cases and controls (additive inheritance model).

## References

[1] Sun H., Saeedi P., Karuranga S. (2022). IDF Diabetes Atlas: Global, Regional and Country-Level Diabetes Prevalence Estimates for 2021 and Projections for 2045. *Diabetes Research and Clinical Practice*.

[2] Yun J. S., Ko S. H. (2021). Current Trends in Epidemiology of Cardiovascular Disease and Cardiovascular Risk Management in Type 2 Diabetes. *Metabolism*.

[3] Krueger S. K., Williams D. E. (2005). Mammalian Flavin-Containing Monooxygenases: Structure/Function, Genetic Polymorphisms and Role in Drug Metabolism. *Pharmacology & Therapeutics*.

[4] Hernandez D., Janmohamed A., Chandan P., Phillips I. R., Shephard E. A. (2004). Organization and Evolution of the Flavin-Containing Monooxygenase Genes of Human and Mouse: Identification of Novel Gene and Pseudogene Clusters. *Pharmacogenetics*.

[5] Lang D. H., Yeung C. K., Peter R. M. (1998). Isoform Specificity of Trimethylamine N-Oxygenation by Human Flavin-Containing Monooxygenase (FMO) and P450 Enzymes: Selective Catalysis by FMO3. *Biochemical Pharmacology*.

[6] Phillips I. R., Dolphin C. T., Clair P. (1995). The Molecular Biology of the Flavin-Containing Monooxygenases of Man. *Chemico-Biological Interactions*.

[7] Phillips I. R., Shephard E. A. (2020). Flavin-Containing Monooxygenase 3 (FMO3): Genetic Variants and Their Consequences for Drug Metabolism and Disease. *Xenobiotica*.

[8] Miao J., Ling A. V., Manthena P. V. (2015). Flavin-Containing Monooxygenase 3 as a Potential Player in Diabetes-Associated Atherosclerosis. *Nature Communications*.

[9] Canyelles M., Tondo M., Cedó L., Farràs M., Escolà-Gil J. C., Blanco-Vaca F. (2018). Trimethylamine N-Oxide: A Link Among Diet, Gut Microbiota, Gene Regulation of Liver and Intestine Cholesterol Homeostasis and HDL Function. *International Journal of Molecular Sciences*.

[10] Wu X., Li C., Mariyam Z. (2018). Acrolein-Induced Atherogenesis by Stimulation of Hepatic Flavin Containing Monooxygenase 3 and a Protection From Hydroxytyrosol. *Journal of Cellular Physiology*.

[11] Yu N., Gu N., Wang Y. (2022). The Association of Plasma Trimethylamine N-Oxide With Coronary Atherosclerotic Burden in Patients With Type 2 Diabetes Among a Chinese North Population. *Diabetes, Metabolic Syndrome and Obesity: Targets and Therapy*.

[12] Shephard E. A., Treacy E. P., Phillips I. R. (2015). Clinical Utility Gene Card for: Trimethylaminuria-Update 2014. *European Journal of Human Genetics*.

[13] Bushueva O., Solodilova M., Churnosov M., Ivanov V., Polonikov A. (2014). The Flavin-Containing Monooxygenase 3 Gene and Essential Hypertension: The Joint Effect of Polymorphism E158K and Cigarette Smoking on Disease Susceptibility. *International Journal of Hypertension*.

[14] Türkanoğlu Özçelik A., Can Demirdöğen B., Demirkaya Ş., Adalı O. (2013). Flavin Containing Monooxygenase 3 Genetic Polymorphisms Glu158Lys and Glu308Gly and Their Relation to Ischemic Stroke. *Gene*.

[15] Morandi A., Zusi C., Corradi M. (2018). Minor Diplotypes of FMO3 Might Protect Children and Adolescents From Obesity and Insulin Resistance. *International Journal of Obesity*.

[16] Alberti K. G., Zimmet P. Z. (1998). Definition, Diagnosis and Classification of Diabetes Mellitus and Its Complications. Part 1: Diagnosis and Classification of Diabetes Mellitus Provisional Report of a WHO Consultation. *Diabetic Medicine*.

[17] Min J. K., Dunning A., Lin F. Y. (2011). Age- and Sex-Related Differences in All-Cause Mortality Risk Based on Coronary Computed Tomography Angiography Findings Results From the International Multicenter CONFIRM (Coronary CT Angiography Evaluation for Clinical Outcomes: An International Multicenter Registry) of 23,854 Patients Without Known Coronary Artery Disease. *Journal of the American College of Cardiology*.

[18] Wang Y., Tong L., Gu N. (2022). Association of Sirtuin 1 Gene Polymorphisms With the Risk of Coronary Heart Disease in Chinese Han Patients With Type 2 Diabetes Mellitus. *Journal of Diabetes Research*.

[19] Goodarzi M. O. (2018). Genetics of Obesity: What Genetic Association Studies Have Taught Us About the Biology of Obesity and Its Complications. *Lancet Diabetes & Endocrinology*.

[20] Zhou M., Wang H., Zhu J. (2016). Cause-Specific Mortality for 240 Causes in China During 1990-2013: A Systematic Subnational Analysis for the Global Burden of Disease Study 2013. *The Lancet*.

[21] Shih D. M., Wang Z., Lee R. (2015). Flavin Containing Monooxygenase 3 Exerts Broad Effects on Glucose and Lipid Metabolism and Atherosclerosis. *Journal of Lipid Research*.

[22] Xu M., Bhatt D. K., Yeung C. K. (2017). Genetic and Nongenetic Factors Associated With Protein Abundance of Flavin-Containing Monooxygenase 3 in Human Liver. *Journal of Pharmacology and Experimental Therapeutics*.

[23] Meijboom W. B., Meijs M. F., Schuijf J. D. (2008). Diagnostic Accuracy of 64-Slice Computed Tomography Coronary Angiography: A Prospective, Multicenter, Multivendor Study. *Journal of the American College of Cardiology*.

