# Supplementary material for: Flavin-Containing Monooxygenase 3 Genetic Variants and Possible Susceptibility to Coronary Heart Disease Among Han Chinese With Type 2 Diabetes
Source: Int J Endocrinol. 2025 Sep 23;2025:1020054. doi: 10.1155/ije/1020054 (PMC12483734; doi:10.1155/ije/1020054)
Supplement: Supporting Information — Additional supporting information can be found online in the Supporting Information section. [file 1020054.f1.docx]

**Supporting Information**


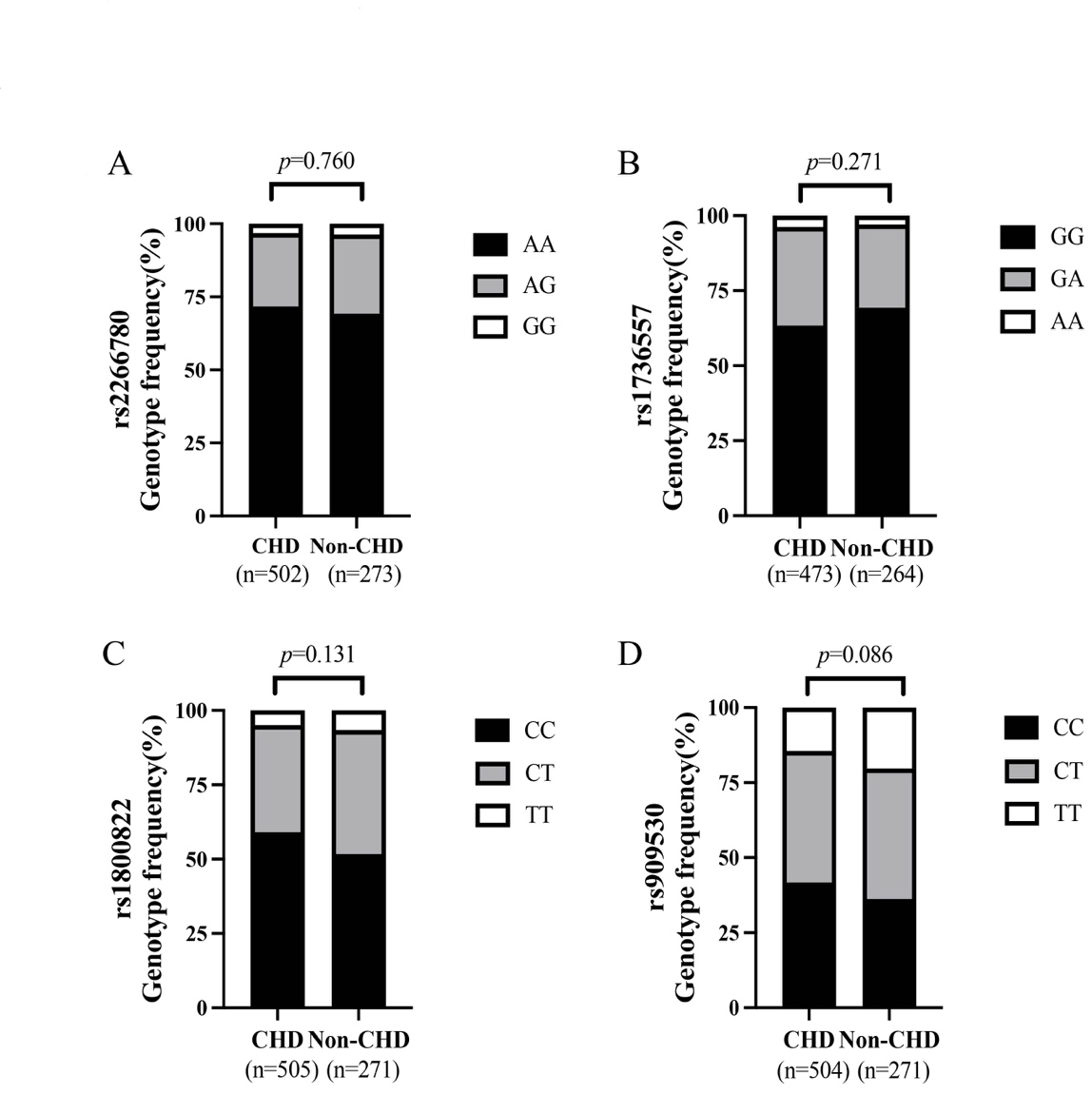


**Figure. S1.** Association analysis of FMO3 candidate SNP genotype frequencies in the patients and control participants.

**Table S1.** Association analysis of FMO3 candidate SNP genotypes in the patients and control participants (recessive inheritance model).

| **SNP** | **Genotype** | **CHD n (%)** | **Non-CHD n (%)** | **OR (95% CI)** | ***P* value** | **OR’ (95%CI’)** | ***P’*-value** |
| --- | --- | --- | --- | --- | --- | --- | --- |
| rs2266780 | AA+AG | 486 (96.8) | 263 (96.3) | 1.000 |  | 1.000 |  |
|  | GG | 16 (3.2) | 10 (3.7) | 1.155 (0.517-2.581) | 0.725 | 1.116 (0.310-4.022) | 0.867 |
| rs1736557 | GG+GA | 455 (96.2) | 256 (97.0) | 1.000 |  | 1.000 |  |
|  | AA | 18 (3.8) | 8 (3) | 0.790 (0.339-1.842) | 0.584 | 0.901 (0.163-4.965) | 0.904 |
| rs1800822 | CC+CT | 480 (95.0) | 253 (93.4) | 1.000 |  | 1.000 |  |
|  | TT | 25 (5.0) | 18 (6.6) | 1.366 (0.731-2.551) | 0.326 | 0.556 (0.188-1.646) | 0.289 |
| rs909530 | CC+CT | 431 (85.5) | 216 (79.7) | 1.000 |  | 1.000 |  |
|  | TT | 73 (14.5) | 55 (20.3) | 0.665 (0.452-0.979) | 0.038 | 0.690 (0.342-1.394) | 0.301 |

**Table S2.** Association analysis of FMO3 candidate SNP genotypes in the patients and control participants (additive inheritance model).

| **SNP** | **Genotype** | **CHD n (%)** | **Non-CHD n (%)** | **OR (95% CI)** | ***P* value** | **OR’ (95%CI’)** | ***P’*-value** |  |
| --- | --- | --- | --- | --- | --- | --- | --- | --- |
| rs2266780 | AA | 360 (71.7) | 189 (69.2) | 1.000 |  | 1.000 |  |  |
|  | AG | 126 (25.1) | 74 (27.1) | 0.894 (0.638-1.252) | 0.514 | 1.301 (0.638-2.658) | 0.469 |  |
|  | GG | 16 (3.2) | 10 (3.7) | 0.840 (0.374-1.887) | 0.673 | 0.951 (0.247-3.658) | 0.942 |  |
| rs1736557 | | GG | 300 (63.4) | 183 (69.3) | 1.000 |  | 1.000 |  |
|  | GA | 155 (32.8) | 73 (27.7) | 1.295 (0.928-1.808) | 0.128 | 1.885 (0.943-3.768) | 0.073 |  |
|  | AA | 18 (3.8) | 8 (3) | 1.372 (0.585-3.220) | 0.467 | 1.101 (0.196-6.182) | 0.913 |  |
| rs1800822 | CC | 298 (59.0) | 140 (51.7) | 1.000 |  | 1.000 |  |  |
|  | CT | 182 (36.0) | 113 (41.7) | 0.757 (0.556-1.031) | 0.077 | 0.744 (0.402-1.376) | 0.346 |  |
|  | TT | 25 (5.0) | 18 (6.6) | 0.652 (0.345-1.235) | 0.190 | 0.573 (0.181-1.810) | 0.343 |  |
| rs909530 | CC | 210 (41.7) | 98 (36.2) | 1.000 |  | 1.000 |  |  |
|  | CT | 221 (43.8) | 118 (43.5) | 0.874 (0.630-1.213) | 0.421 | 1.202 (0.629-2.295) | 0.577 |  |
|  | TT | 73 (14.5) | 55 (20.3) | 0.619 (0.405-0.974) | 0.027 | 0.765 (0.342-1.713) | 0.515 |  |

**Table S3.** Analysis of haplotype.

|  | | **Haplotype** | |  | |  | **CHD (%)** | | | **Non-CHD (%)** | | | | **OR (95% CI)** | | ***P* value** | |
| --- | --- | --- | --- | --- | --- | --- | --- | --- | --- | --- | --- | --- | --- | --- | --- | --- | --- |
| **rs1800822** | **rs1736557** | | **rs909530** | | **rs2266780** | | | | **n=1012** | **n=550** |  | | | |  | | |
| C | | G | | C | | A | | 413 (40.8) | | 211 (38.4) | | | | 1.000 |  | | |
| T | | G | | T | | A | | 207 (20.5) | | 137 (24.9) | | 0.772 (0.588-1.013) | | | | | 0.062 |
| C | | G | | T | | G | | 160 (15.8) | | 95 (17.2) | | 0.860 (0.635-1.165) | | | | | 0.331 |
| C | | A | | C | | A | | 205 (20.3) | | 92 (16.8) | | 1.138 (0.846-1.532) | | | | | 0.392 |
| T | | G | | C | | A | | 27 (2.5) | | 15 (2.8) | | | 0.920 (0.479-1.766) | | | | 0.801 |

**Table S4．**Association of rs1800822 genotypes with CHD risk in the patients and control participants (dominant inheritance model)

| **SNP** | **Genotype** | **CHD**  **n (%)** | **Non-CHD**  **n (%)** | | **OR (95% CI)** | ***P* value** |
| --- | --- | --- | --- | --- | --- | --- |
| rs1800822 | CC | 298 (59.0) | | 140 (51.7) | 1.000 |  |
| unadjusted | CT+TT | 207 (41.0) | | 131 (48.3) | 0.742(0.552-0.999) | 0.049 |
| Model 1 |  |  | |  | 0.771(0.528-1.127) | 0.180 |
| Model 2 |  |  | |  | 0.826(0.554-1.231) | 0.347 |
| Model 3 |  |  | |  | 0.641(0.366-1.125) | 0.121 |

Note: Model 1: age, sex, smoking status and duration of diabetes; Model 2: age, sex, smoking status, duration

of diabetes and BMI; Model 3: age, sex, smoking status, duration of diabetes, BMI, Hb1Ac, eGFR, LDL-C and HDL-C.

**Table S5．**Association of rs909530 genotypes with CHD risk in the patients and control participants (recessive inheritance model)

| **SNP** | **Genotype** | **CHD**  **n (%)** | **Non-CHD n (%)** | | **OR (95% CI)** | ***P* value** |
| --- | --- | --- | --- | --- | --- | --- |
| rs909530 | CC+CT | 431 (85.5) | 216 (79.7) | 1.000 | |  |
| unadjusted | TT | 73 (14.5) | 55 (20.3) | 0.665 (0.452-0.979) | | 0.038 |
| Model 1 |  |  |  | 0.712(0.438-1.155) | | 0.169 |
| Model 2 |  |  |  | 0.707(0.424-1.177) | | 0.183 |
| Model 3 |  |  |  | 0.690(0.342-1.396) | | 0.301 |

Note: Model 1: age, sex, smoking status and duration of diabetes; Model 2: age, sex, smoking status, duration

of diabetes and BMI; Model 3: age, sex, smoking status, duration of diabetes, BMI, Hb1Ac, eGFR, LDL-C and HDL-C.

**Table S6．**Association of rs909530 genotypes with CHD risk in the patients and control participants (additive inheritance model)

| **SNP** | **Genotype** | | | **CHD**  **n (%)** | **Non-CHD**  **n (%)** | | **OR (95% CI)** | ***P* value** | | |
| --- | --- | --- | --- | --- | --- | --- | --- | --- | --- | --- |
| rs909530 | CC | | 210 (41.7) | | 98 (36.2) | | 1.000 | |  |  |
|  | CT | | 221 (43.8) | | 118 (43.5) | 0.874 (0.630-1.213) | | | 0.421 |  |
| unadjusted | TT | | 73 (14.5) | | 55 (20.3) | 0.619 (0.405-0.974) | | | 0.027 |  |
| Model 1 |  | |  | |  | 0.702(0.414-1.193) | | | 0.191 |  |
| Model 2 |  | |  | |  | | 0.706(0.406-1.229) | | 0.219 |  |
| Model 3 |  |  | | |  | | 0.765(0.342-1.713) | | 0.515 |  |

Note: Model 1: age, sex, smoking status and duration of diabetes; Model 2: age, sex, smoking status, duration

of diabetes and BMI; Model 3: age, sex, smoking status, duration of diabetes, BMI, Hb1Ac, eGFR, LDL-C and HDL-C.

**Table S7.** Clinical characteristics of the cases and controls.

|  | **CHD**  **(n=506)** | **Non-CHD**  **(n=173)** | ***P* value** |
| --- | --- | --- | --- |
| Sex, male n (%) | 323 (63.80) | 72 (41.60) | **＜0.001** |
| Age (years) | 64.93±9.89 | 62.87±9.99 | **＜0.018** |
| Diabetes duration (years) | 8.00 (5.00-15.00) | 8.00 (4.00-16.00) | 0.600 |
| Hypertension n (%) | 378 (74.70) | 128 (73.99) | 0.852 |
| FBG (mmol/l) | 7.24 (6.15-8.83) | 6.86 (6.10-8.40) | 0.204 |
| HbA1c (%) | 7.20 (6.60-8.30) | 6.80 (6.20-7.65) | **0.003** |
| eGFR (ml/min/1.73m^2^) | 75.00±22.56 | 80.34±21.72 | **0.022** |
| TG (mmol/l) | 1.61 (1.08-2.33) | 1.52 (1.07-2.53) | 0.598 |
| TC (mmol/l) | 3.82 (3.27-4.65) | 4.04 (3.48-4.91) | 0.175 |
| LDL-C (mmol/l) | 2.17 (1.70-2.85) | 2.29 (1.77-2.76) | 0.736 |
| HDL-C (mmol/l) | 0.95 (0.83-1.08) | 1.01 (0.88-1.16) | **＜0.007** |
| BMI (kg/㎡) | 26.18±3.67 | 26.91±3.98 | **0.035** |

Note: Continuous data with normal distribution are shown as means ± SD, skewed data as medians (interquartile ranges), and categorical variables as counts (%).

**Table S8.** Association analysis of FMO3 candidate SNP genotypes in cases and controls (dominant inheritance model).

| **SNP** | **Genotype** | **CHD n (%)** | **Non-CHD n (%)** | **OR (95% CI)** | ***P* value** | **OR’ (95%CI’)** | ***P’*-value** |
| --- | --- | --- | --- | --- | --- | --- | --- |
| rs2266780 | AA | 360(71.7) | 118 (60.0) | 1.000 |  | 1.000 |  |
|  | AG+GG | 142 (28.3) | 53 (40.0) | 0.878 (0.602-1.281) | 0.500 | 1.241 (0.669-2.320) | 0.489 |
| rs1736557 | GG | 300 (63.4) | 111 (68.1) | 1.000 |  | 1.000 |  |
|  | GA+AA | 173 (36.6) | 52 (31.9) | 1.231 (0.843-1.798) | 0.282 | 1.716 (0.914-3.223) | 0.093 |
| rs1800822 | CC | 298 (59.0) | 88 (51.8) | 1.000 |  | 1.000 |  |
|  | CT+TT | 207 (41.0) | 82 (48.2) | 0.745 (0.526-1.057) | 0.099 | 0.641 (0.366-1.125) | 0.121 |
| rs909530 | CC | 210 (41.7) | 63 (37.1) | 1.000 |  | 1.000 |  |
|  | CT+TT | 294 (58.3) | 107 (62.9) | 0.824 (0.576-1.179) | 0.290 | 0.961 (0.546-1.691) | 0.889 |

**Table S9.** Association analysis of FMO3 candidate SNP genotypes in cases and controls (recessive inheritance model).

| **SNP** | **Genotype** | **CHD n (%)** | **Non-CHD n (%)** | **OR (95% CI)** | ***P* value** | **OR’ (95%CI’)** | ***P’*-value** |
| --- | --- | --- | --- | --- | --- | --- | --- |
| rs2266780 | AA+AG | 486 (96.8) | 162 (94.7) | 1.000 |  | 1.000 |  |
|  | GG | 16 (3.2) | 9 (5.3) | 0.593 (0.257-1.367) | 0.220 | 1.116 (0.310-4.022) | 0.867 |
| rs1736557 | GG+GA | 455 (96.2) | 158 (96.9) | 1.000 |  | 1.000 |  |
|  | AA | 18 (3.8) | 5 (3.1) | 1.250 (0.457-3.423) | 0.664 | 0.901 (0.163-4.965) | 0.904 |
| rs1800822 | CC+CT | 480 (95.0) | 158 (92.9) | 1.000 |  | 1.000 |  |
|  | TT | 25 (5.0) | 12 (7.1) | 0.686 (0.337-1.397) | 0.299 | 0.556 (0.188-1.646) | 0.289 |
| rs909530 | CC+CT | 431 (85.5) | 132 (77.6) | 1.000 |  | 1.000 |  |
|  | TT | 73 (14.5) | 38 (22.4) | 0.588 (0.380-0.912) | 0.018 | 0.690 (0.342-1.394) | 0.301 |

**Table S10.** Association analysis of FMO3 candidate SNP genotypes in cases and controls (additive inheritance model).

| **SNP** | **Genotype** | **CHD n (%)** | **Non-CHD n (%)** | **OR (95% CI)** | ***P* value** | **OR’ (95%CI’)** | ***P’*-value** |  |
| --- | --- | --- | --- | --- | --- | --- | --- | --- |
| rs2266780 | AA | 360 (71.7) | 118 (69.0) | 1.000 |  | 1.000 |  |  |
|  | AG | 126 (25.1) | 44 (25.7) | 0.939 (0.628-1.402) | 0.757 | 1.258 (0.647-2.447) | 0.499 |  |
|  | GG | 16 (3.2) | 9 (5.3) | 0.583 (0.251-1.353) | 0.209 | 1.188 (0.325-4.343) | 0.795 |  |
| rs1736557 | | GG | 300 (63.4) | 111 (68.1) | 1.000 |  | 1.000 |  |
|  | GA | 155 (32.8) | 47 (28.8) | 1.220 (0.824-1.806) | 0.320 | 1.794 (0.934-3.447) | 0.079 |  |
|  | AA | 18 (3.8) | 5 (3.1) | 1.332 (0.483-3.673) | 0.580 | 1.081 (0.194-6.033) | 0.930 |  |
| rs1800822 | CC | 298 (59.0) | 88 (51.8) | 1.000 |  | 1.000 |  |  |
|  | CT | 182 (36.0) | 70 (41.2) | 0.768 (0.534-1.105) | 0.155 | 0.678 (0.374-1.227) | 0.199 |  |
|  | TT | 25 (5.0) | 12 (7) | 0.615 (0.297-1.274) | 0.191 | 0.483 (0.160-1.460) | 0.197 |  |
| rs909530 | CC | 210 (41.7) | 63 (37.1) | 1.000 |  | 1.000 |  |  |
|  | CT | 221 (43.8) | 69 (40.6) | 0.961 (0.650-1.420) | 0.841 | 1.094 (0.589-2.032) | 0.776 |  |
|  | TT | 73 (14.5) | 38 (22.3) | 0.576 (0.356-0.934) | 0.025 | 0.722 (0.335-1.557) | 0.406 |  |
